# Transcriptomic Analysis of Brown Adipose Tissue across the Physiological Extremes of Natural Hibernation

**DOI:** 10.1371/journal.pone.0085157

**Published:** 2013-12-30

**Authors:** Marshall Hampton, Richard G. Melvin, Matthew T. Andrews

**Affiliations:** 1 Department of Mathematics and Statistics, University of Minnesota Duluth, Duluth, Minnesota, United States of America; 2 Department of Biology, University of Minnesota Duluth, Duluth, Minnesota, United States of America; Karolinska Institutet, Sweden

## Abstract

We used RNAseq to generate a comprehensive transcriptome of Brown Adipose Tissue (BAT) over the course of a year in the naturally hibernating thirteen-lined ground squirrel, *Ictidomys tridecemlineatus*. During hibernation ground squirrels do not feed and use fat stored in White Adipose Tissue (WAT) as their primary source of fuel. Stored lipid is consumed at high rates by BAT to generate heat at specific points during the hibernation season. The highest rate of BAT activity occurs during periodic arousals from hypothermic torpor bouts, referred to as Interbout Arousals (IBAs). IBAs are characterized by whole body re-warming (from 5 to 37 °C) in 2-3 hours, and provide a unique opportunity to determine the genes responsible for the highly efficient lipid oxidation and heat generation that drives the arousal process. Illumina HighSeq sequencing identified 14,573 distinct BAT mRNAs and quantified their levels at four points: active ground squirrels in April and October, and hibernating animals during both torpor and IBA. Based on significant changes in mRNA levels across the four collection points, 2,083 genes were shown to be differentially expressed. In addition to providing detail on the expression of nuclear genes encoding mitochondrial proteins, and genes involved in beta-adrenergic and lipolytic pathways, we identified differentially expressed genes encoding various transcription factors and other regulatory proteins which may play critical roles in high efficiency fat catabolism, non-shivering thermogenesis, and transitions into and out of the torpid state.

## Introduction

Almost all mammals possess Brown Adipose Tissue (BAT) as infants to help maintain their body temperature through nonshivering thermogenesis. Unlike White Adipose Tissue (WAT), which serves as the main fat storage depot in children and adults, BAT has evolved to generate heat in newborn and hibernating mammals (reviewed in [1]). Mammals that hibernate retain BAT as adults in order to efficiently re-warm themselves when arousing from hypothermic torpor. The discovery of BAT in adult humans [2-5] has generated excitement in the biomedical community because of its potential for accelerating weight loss in obese individuals [6-8]. However very little is known about the genes required for optimal BAT function.

**Figure 1:**
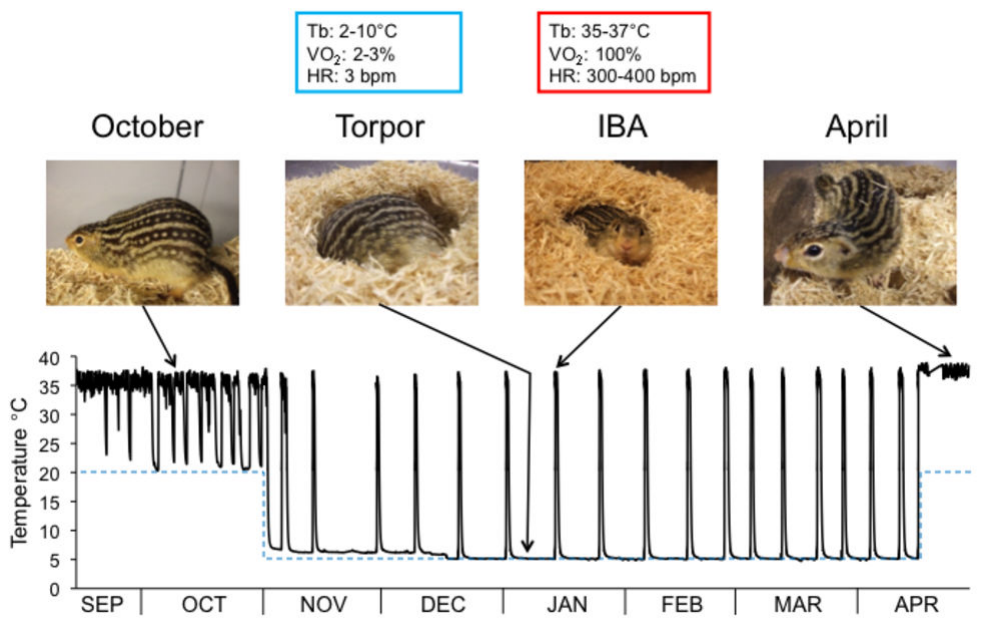
Graph showing body temperature tracings (solid line) of a thirteen-lined ground squirrel inside an environmental chamber beginning in September and ending in April. Body temperature was measured using a surgically implanted transmitter. The dashed blue line represents the ambient (environmental) temperature, which is lowered to 5°C on November 1 and raised back to 23°C in March or April depending on the experiment. Periodic interbout arousals (IBAs) are seen as regular spikes in body temperature despite a constant ambient temperature of 5°C. Photographs of animals at four different points during the year are indicated and shown above the graph [70]. Characteristic measurements of body temperature (T_b_), oxygen consumption (VO_2_), and heart rate (HR) during torpor and IBA are shown above the respective photographs.

In this study we examined BAT gene expression in a naturally hibernating mammal, the thirteen-lined ground squirrel (*Ictidomys tridecemlineatus*). During 5 months of hibernation these animals do not eat and use fat stores as their primary source of fuel. Stored lipid is consumed at high rates by BAT to generate heat at specific points during the hibernation season. The highest rate of BAT activity occurs during multiple arousals from hypothermic torpor bouts referred to as Interbout Arousals (IBAs). IBAs occur every 1-2 weeks and are characterized by rapid whole body re-warming and a resumption of normothermic metabolic activity for 12-24 hours (Figure 1). IBAs also provide a unique opportunity to determine the molecular factors in BAT that are responsible for the highly efficient fat burning activity that drives the arousal process. During the 2-3 hour transition out of torpor, O_2_ consumption increases 50-fold, heart rate explodes from less than 10 beats per minute (bpm) to over 400 bpm, and body temperature increases from 5 to 37°C [9]. 

Lipid catabolism by BAT in natural hibernators is likely the highest capacity oxidation of fat (per mass of tissue) that occurs in mammals. With multiple mitochondria that uncouple the electron transport chain from ATP synthesis, and a high density of capillaries to deliver O_2_, BAT has evolved to maximize the combustion of fat to generate heat in a short amount of time [1]. Despite the inherent advantages of knowing how this fat-burning machine works, there is little information on the genes that control this process. In this study we identified genes that are expressed in BAT by purifying BAT RNA at four distinct phases of the hibernation season followed by Illumina sequencing of the corresponding cDNAs to profile gene expression at each phase. 

## Results

Total RNA was prepared from BAT during various activity states at four different times of the year: April active, October active, January torpid, and interbout arousal (Figure 1). Illumina HighSeq 2000 sequencing of cDNAs derived from 24 animals resulted in 179,979,787 high quality reads of 100 bases each. For comparison purposes we also obtained a total of 110,399,607 high quality reads of 76 bases each from WAT using the Illumina GAII platform. The BAT sequence datasets were submitted to the Sequence Read Archive under accession SRS396817.

Sequence reads were mapped to contigs constructed from this project and other Illumina sequence reads from thirteen-lined ground squirrel skeletal muscle and heart tissue, and from hypothalamus and brain cortex [10]. A preliminary set of contigs was assembled using the Trinity suite of programs [11]. This assembly produced 184,609 contigs of a mean length of 1,133 bases. Trinity was also used to predict coding domain subsequences from these contigs. Any contig containing a predicted coding domain was then trimmed to this domain plus up to 100 bases on both ends of the domain. This resulted in a set of 81,978 contigs with mean length 1,037 bases which were used for protein-coding gene identification. These contigs were then compared to the NCBI RefSeq human mRNA sequences using NCBI Blastn [12]. In total, we identified 14,573 distinct transcripts in ground squirrel BAT that mapped to human mRNAs (Table S1). Before matching raw reads to the contigs, mitochondrial-encoded reads were screened using the *I. tridecemlineatus* mitochondrial sequence that we assembled previously [13] and NCBI's megablast program [12]. Transcripts from the mitochondrial genome comprised approximately 40% of the total BAT reads, which is a far greater percentage than seen in other ground squirrel tissues (Table 1).

**Table 1:** Percentage of RNA reads from ground squirrel mitochondrial genome.

	April	October	Torpor	IBA
Skeletal Muscle	20	14	14	12
Hypothalamus	8	7	6	6
WAT	3	3	3	3
BAT	35	44	40	40

**Figure 2:**
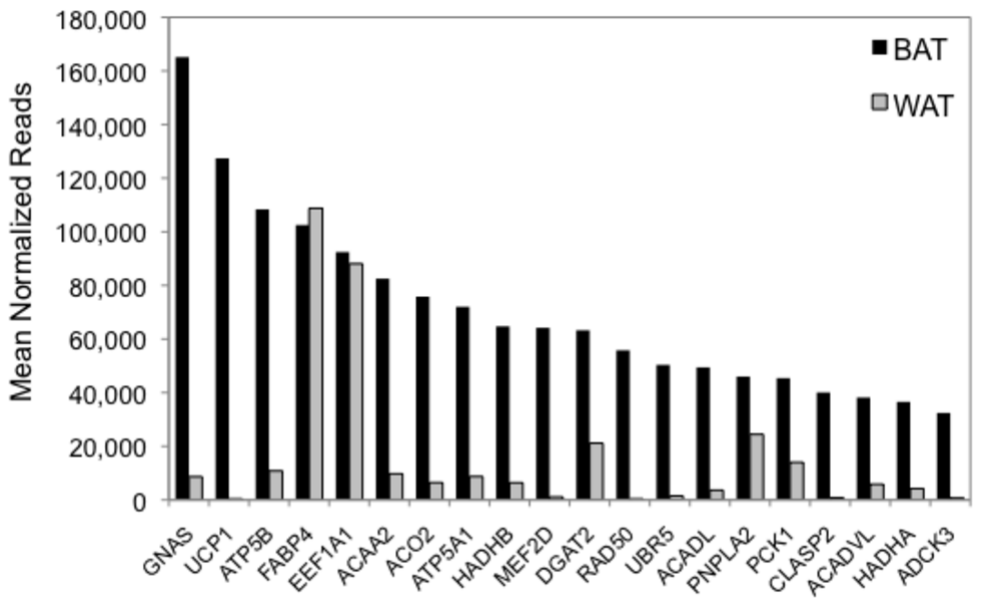
Twenty most abundant mRNAs in ground squirrel BAT (black) shown along with their respective levels in WAT (gray). Measurements shown on the x-axis are the mean of the upper-quartile normalized counts of reads from the four collection points. Abbreviations: *ACAA2*, acetyl-CoA acyltransferase 2; ACADL, acyl-CoA dehydrogenase, long chain; ACADVL, acyl-CoA dehydrogenase, very long chain; *ADCK3*, aarF domain containing kinase 3; ACO2, aconitase 2; ATP5A1, ATP synthase, alpha subunit 1; ATP5B, ATP synthase, beta polypeptide; CLASP2, cytoplasmic linker associated protein 2; *DGAT2*, diacylglycerol O-acyltransferase 2; EEF1A1, eukaryotic translation elongation factor 1 alpha 1; *FABP4*, fatty acid binding protein 4; GNAS, G-protein subunit G_s_α; HADHA and HADHB, hydroxyacyl-CoA dehydrogenase/3-ketoacyl-CoA thiolase/enoyl-CoA hydratase (trifunctional protein), alpha subunit and beta subunit; MEF2D, myocyte enhancer factor 2D; PCK1, phosphoenolpyruvate carboxykinase 1; PNPLA2, patatin-like phospholipase domain containing protein 2; RAD50, RAD50 homolog (*S. cerevisiae*); UBR5, ubiquitin protein ligase E3 component n-recognin 5; UCP1, uncoupling protein 1.

**Figure 3:**
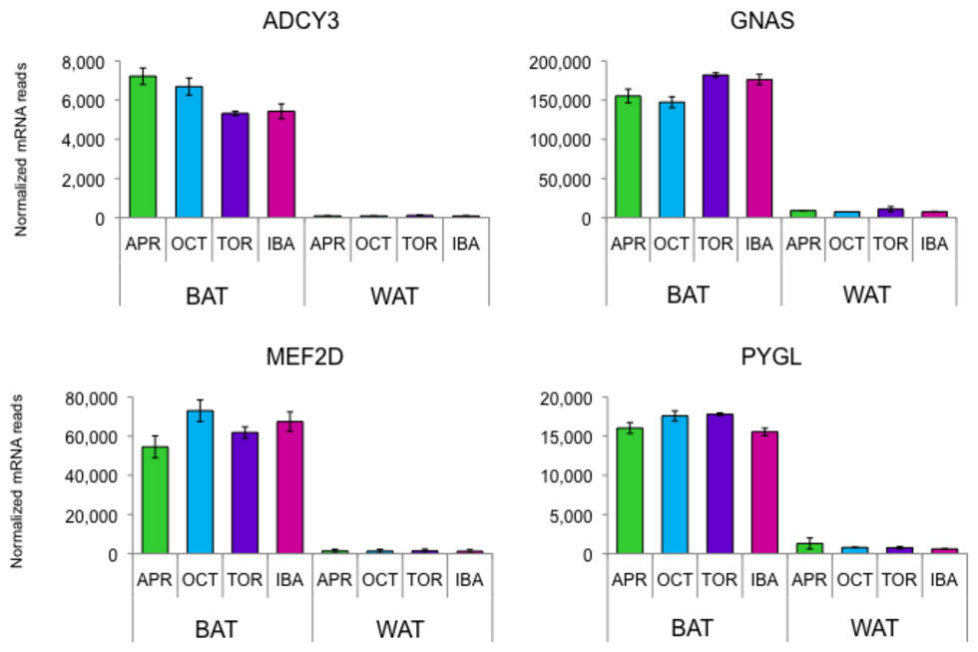
Seasonal expression of four genes that are highly enriched in BAT versus WAT. Error bars represent standard error of the mean. *Abbreviations: ADCY3*, Adenylate cyclase 3; GNAS, G-protein subunit G_s_α; MEF2D, myocyte enhancer factor 2D; PYGL, phosphorylase, glycogen, liver.

The twenty most highly expressed BAT transcripts based on the mean of the upper-quartile normalized counts of reads from the four collection points are shown in Figure 2 along with their related counts in WAT. Most of these are mitochondrial-targeted proteins or involved in beta-adrenergic and lipolytic pathways. Figure 3 shows the seasonal expression of four genes that are highly enriched in BAT versus WAT including the massively expressed G-protein subunit G_s_α (*GNAS*), transcription factor *MEF2D*, adenylate cyclase 3 (*ADCY3*), and phosphorylase, glycogen, liver (*PYGL*).

To determine genes that are differentially expressed across the four collection points, the test statistic p-values computed by DESeq for each set of genes were independently filtered [14] by restricting to those with a mean of at least 100 normalized counts at one collection point, and with a 50% change between the means of any two collection points. After this filtering, there were 2,631 candidate genes that were differentially expressed in BAT. The Benjamini-Hochberg algorithm [15] with a false discovery rate of 0.05 was used to select the final set of 2,083 differentially expressed genes in BAT (Table S2). 

### Mitochondrial-targeted proteins

Due to the abundance of mitochondria in BAT, the transcriptome is greatly enriched in mRNAs from the mitochondrial genome (Table 1) and the nuclear genome encoding mitochondrial-targeted proteins. Out of 835 nuclear genes identified as coding for mitochondrial proteins, 170 were differentially expressed in BAT. Table 2 shows some of the differentially expressed genes encoding mitochondrial proteins such as the thermogenic uncoupling protein 1 (*UCP1*), acyl-CoA synthetase long-chain family member 1 (*ACSL1*), pyruvate dehydrogenase kinase, isozyme 4 (*PDK4*), carnitine/acylcarnitine translocase (*SLC25A20*), and carnitine palmitoyltransferase 1A (*CPT1A*) and 2 (*CPT2*).

**Table 2:** Differentially expressed nuclear genes encoding BAT mitochondrial proteins showing highest expression in October, Torpor, or IBA.

Gene	Name/description	Fold change over April	Number of normalized reads			
			April	October	Torpor	IBA
KMO	kynurenine 3-monooxygenase (kynurenine 3-hydroxylase)	12.2	48	96	454	587
UCP3	uncoupling protein 3	6.0	153	131	690	923
ABCD3	ATP-binding cassette, sub-family D (ALD), member 3	2.3	4602	3808	10708	8333
MCART1	mitochondrial carrier triple repeat 1	2.3	1889	4085	4316	4232
ACSL1	acyl-CoA synthetase long-chain family member 1	2.2	14295	20827	30772	27682
DECR1	2,4-dienoyl CoA reductase 1	2.2	6414	14081	12121	10706
PREP	prolyl endopeptidase	2.2	690	1235	1521	1545
UCP1	uncoupling protein 1	2.2	74907	111962	165776	156951
SLC25A20	solute carrier family 25 (carnitine/acylcarnitine translocase), member 20	2.1	4923	8229	10021	10367
ACADM	acyl-CoA dehydrogenase, C-4 to C-12 straight chain	2.0	12577	24105	23427	24740
MARCH5	membrane-associated ring finger (C3HC4) 5	2.0	862	1376	1701	1735
TIMM50	translocase of inner mitochondrial membrane 50 homolog (S. cerevisiae)	2.0	1041	1601	2045	1896
CPT1A	carnitine palmitoyltransferase 1A (liver)	1.9	11994	14985	22769	20277
HADHA	hydroxyacyl-CoA dehydrogenase/3-ketoacyl-CoA thiolase/enoyl-CoA hydratase (trifunctional protein), alpha subunit	1.8	25333	36671	44564	39719
HADHB	hydroxyacyl-CoA dehydrogenase/3-ketoacyl-CoA thiolase/enoyl-CoA hydratase (trifunctional protein), beta subunit	1.8	42827	65428	75912	74762
PDK4	pyruvate dehydrogenase kinase, isozyme 4	1.7	5968	1575	10244	9716
PINK1	PTEN induced putative kinase 1	1.7	2368	4071	3069	3150
SUCLA2	succinate-CoA ligase, ADP-forming, beta subunit	1.7	11440	18296	19453	18268
CPT2	carnitine palmitoyltransferase 2	1.6	6166	7619	9973	9584
CRLS1	cardiolipin synthase 1	1.5	4082	5511	6322	5866
ETFDH	electron-transferring-flavoprotein dehydrogenase	1.5	11969	16228	18390	16391

**Figure 4:**
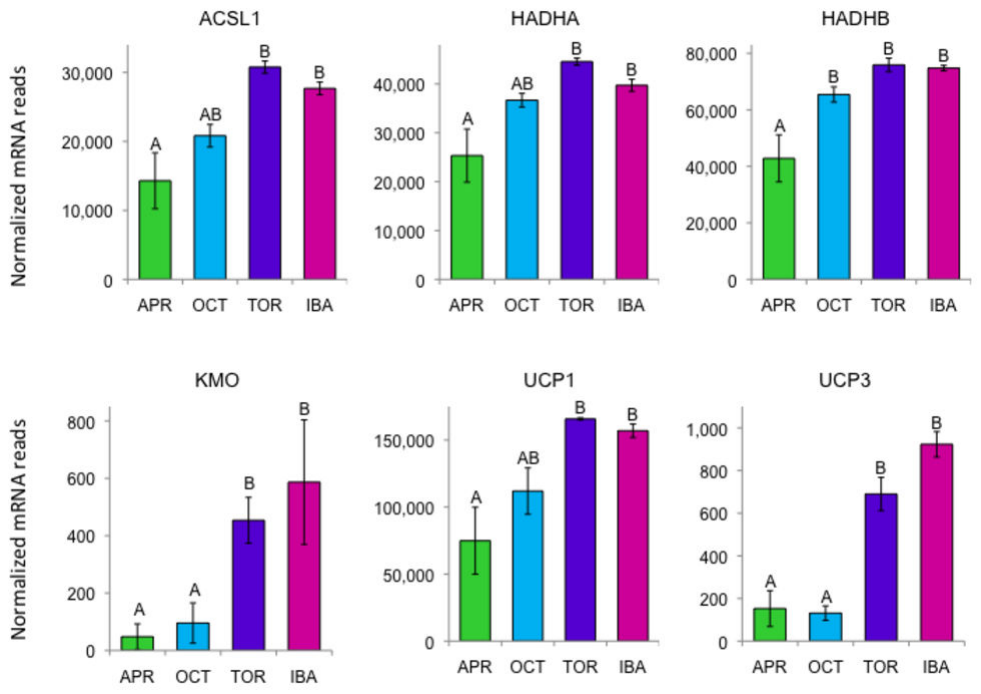
Differentially expressed genes encoding representative mitochondrial proteins in BAT. Error bars represent standard error of the mean. Transcript levels for all genes shown have an FDR<0.05. The letters above each bar represent post hoc pair-wise comparisons to determine significance between collection points. Any collection point not connected by the same letter is significantly different (FDR<0.05). *Abbreviations: ACSL1*, acyl-CoA synthetase long-chain family member 1; HADHA and HADHB, hydroxyacyl-CoA dehydrogenase/3-ketoacyl-CoA thiolase/enoyl-CoA hydratase (trifunctional protein), alpha subunit and beta subunit; *KMO*, kynurenine 3-monooxygenase; UCP1 and UCP3, uncoupling protein 1 and 3.

Examples of differentially expressed genes coding for mitochondrial proteins that are more highly expressed during torpor and/or IBA are shown in Figure 4. One of the most dramatic of these is *KMO* (kynurenine 3-monooxygenase), whose expression is ten times higher in torpor and IBA compared to April. KMO catalyzes the first step in the conversion of kynurenine to quinolinic acid - a precursor of the redox cofactor nicotinamide adenine dinucleotide (NAD) used by the mitochondrial electron transport chain (reviewed in 16). Expression of *UCP1* and *UCP3* are also highlighted in Figure 4. The 100-fold higher level of transcripts for UCP1 versus UCP3 is consistent with previous results in BAT from Arctic ground squirrels where UCP1 mRNA is highly abundant, but UCP3 mRNA could not be detected [17]. 

### Adrenergic signaling

**Figure 5:**
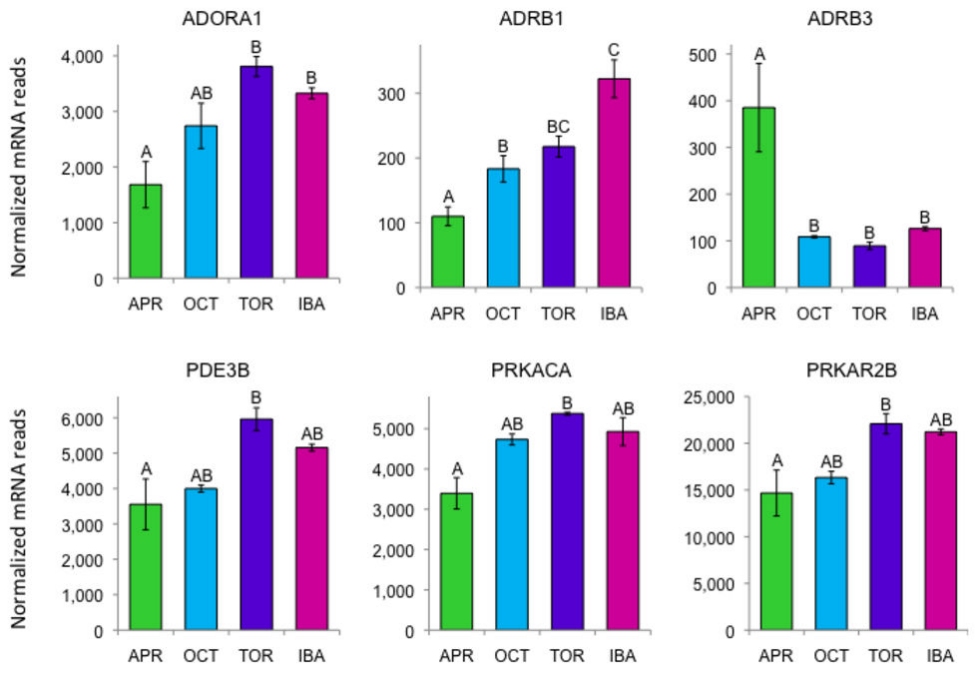
Differentially expressed genes encoding representative proteins in the adrenergic signaling pathway in BAT. Error bars represent standard error of the mean. Transcript levels for all genes shown have an FDR<0.05. The letters above each bar represent post hoc pair-wise comparisons to determine significance between collection points. Any collection point not connected by the same letter is significantly different (FDR<0.05). *Abbreviations: ADORA1*, adenosine A1 receptor; *ADBR1* and *ADBR3*, beta-adrenergic receptors 1 and 3; *PDE3B*, phosphodiesterase 3B, cGMP-inhibited; PRKACA, protein kinase A catalytic subunit; PRKAR2B, protein kinase A, cAMP-dependent, regulatory subunit, type II, beta.

Adrenergic signaling controls high-throughput lipolysis and ultimately heat-generation in BAT (reviewed in [1]). Beta-adrenergic receptor genes *ADRB1* and *ADRB3* show opposite patterns of expression, with *ADRB3* much higher in April (Figure 5). Changes in hibernator adrenergic receptor type are discussed in Kramarova et al. [18]. The beta-adrenergic receptors are coupled to the G-protein stimulatory subunit GNAS. Although not differentially expressed, *GNAS* was expressed at extremely high levels at all time points (Figure 3) with the highest number of reads of any mRNA in BAT. Activation of natriuretic peptide receptors can induce effects similar to the beta-adrenergic pathway [19]. It is interesting that all three natriuretic peptide receptor genes are expressed in BAT (*NPR1*, *NPR2*, *NPR3*) at levels similar to the beta-adrenergic receptor genes, but only *NPR3* is differentially expressed, with higher values in torpor and IBA (Table S2).

Once activated by the beta-adrenergic receptors, GNAS stimulates the production of cAMP by activating membrane-bound adenylate cyclases. We found several types of adenylate cyclase genes are expressed in BAT: *ADCY3* (62% of ADCY mRNAs), *ADCY6* (24%), *ADCY4* (5%), *ADCY9* (5%), *ADCY10* (2%), and very small transcript levels of *ADCY5*, *ADCY7*, *ADCY8*, and *ADCY2*. Only *ADCY4* is differentially expressed, with the highest expression in April. *ADCY3* is very BAT specific (Figure 3), with low expression in the other tissues available for comparison (heart, skeletal muscle, WAT, hypothalamus, and cortex).

 The cAMP produced by the adenylate cyclases activates protein kinase A, which is composed of the catalytic subunit PRKACA and the regulatory subunit PRKAR2B. Genes for both of these protein kinase A subunits are differentially expressed with highest levels of expression in torpor and IBA (Figure 5). cAMP is converted back to AMP by phosphodiesterases. Several phosphodiesterase genes were differentially expressed in BAT, most abundant of which were *PDE3B* (phosphodiesterase 3B, cGMP-inhibited; Figure 5), and *PDE8A* (phosphodiesterase 8A). The adenosine A1 receptor *ADORA1* has its highest expression in torpor and lowest in April (Figure 5). ADORA1 inhibits adenylate cyclase [20] and may down-regulate non-shivering thermogenesis by slowing adrenergic signaling during torpor. Recently, central activation of ADORA1 in cold-exposed (15 °C) rats was shown to inhibit BAT thermogenesis and result in a hypothermic torpor-like state [21].

### Lipolysis, glycogen utilization, and lipid transport

**Figure 6:**
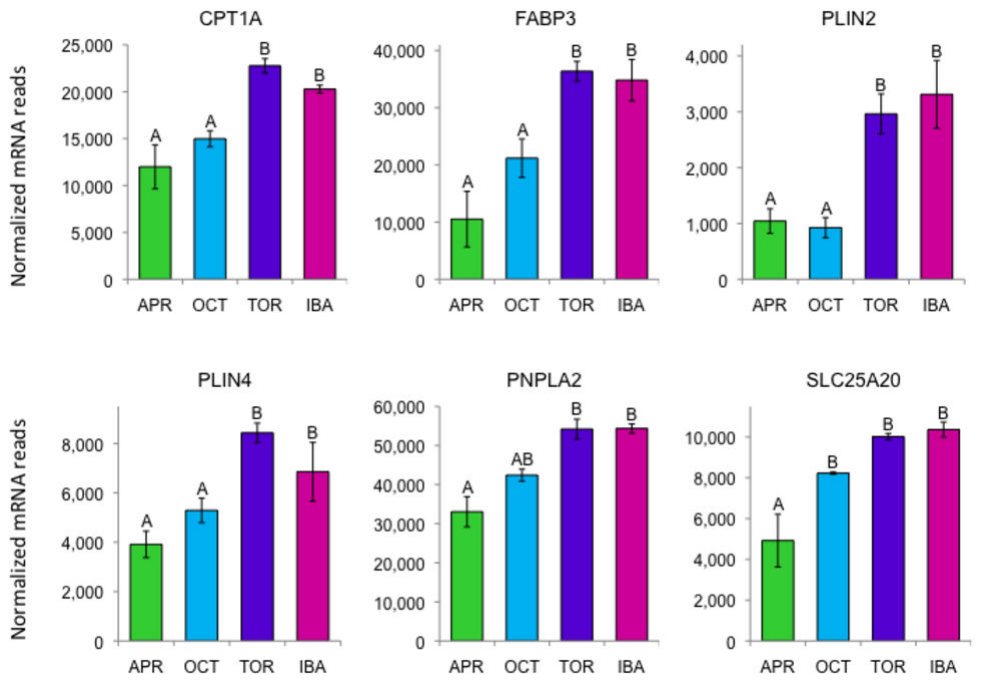
Differentially expressed genes encoding representative proteins involved in lipolysis or fatty acid transport in BAT. Error bars represent standard error of the mean. Transcript levels for all genes shown have an FDR<0.05. The letters above each bar represent post hoc pair-wise comparisons to determine significance between collection points. Any collection point not connected by the same letter is significantly different (FDR<0.05). Abbreviations: *CPT1A*, carnitine palmitoyltransferase 1A; FABP3, fatty acid binding protein 3; *PLIN2* and *PLIN4*, perilipin 2 and 4; PNPLA2, patatin-like phospholipase domain containing protein 2; *SLC25A20*, mitochondrial carnitine/acylcarnitine translocase.

The catalytic subunit of protein kinase A, PRKACA, phosphorylates and activates PYGL (glycogen phosphorylase, liver), PNPLA2 (patatin-like phospholipase domain containing 2, also known as ATGL) and LIPE (hormone sensitive lipase). *PYGL* transcripts are abundant (Figure 3) although not differentially expressed. PYGL breaks down glycogen to produce glucose, which is required to support increased nonshivering thermogenesis in BAT [22]. PNPLA2 liberates the first free fatty acid from triglycerides, and the *PNPLA2* gene is differentially expressed with highest values in torpor and IBA (Figure 6). LIPE cleaves the next fatty acid from the diacylglycerol; it has high mRNA levels in BAT, but is not differentially expressed.

Transcripts for perilipins PLIN2, PLIN4, and PLIN5, which regulate the access of PNPLA2 and LIPE to triacylglycerol (TAG) droplets [23,24], are abundant in BAT and differentially expressed, with higher expression in torpor and IBA. In addition, *PLIN1* mRNA is abundant in BAT, but is not differentially expressed. The strong expression of *PLIN4* (Figure 6) is notable considering its low expression in BAT in mice [25].

 Once free fatty acids are cleaved from TAG they must be transported to the mitochondria. Fatty acid binding protein *FABP3* is differentially expressed (Figure 6), with mean expression over three times higher in torpor and IBA than April. *FABP4* was very highly expressed in BAT but did not meet our differential expression criteria. Mitochondrial import of fatty acids can be facilitated by the mitochondrial carnitine/acylcarnitine translocase (*SLC25A20*) and by carnitine palmitoyltransferases *CPT1A* and *CPT2*, all three of which are up-regulated during hibernation (Figure 6).

 In addition to generating free fatty acids from intracellular stores, BAT also utilizes lipids from the circulation. CUBN (cubilin) is a receptor for apolipoprotein-A1 and high-density lipoprotein [26], and albumin [27], so it may be important for lipid import in BAT. *CUBN* is differentially expressed showing much higher levels in October, torpor, and IBA. *OBP2B* (odorant-binding protein 2B), a lipocalin, and *REEP6* (receptor accessory protein 6), which may help locate OBP2B on the membrane [28], are both highly BAT-specific but not differentially expressed. These proteins may also be associated with endocytotic lipid import in BAT. The related *REEP4* is differentially expressed but at relatively low levels. In this context it is worth noting that the gene coding CD36, a protein known to be important in fatty acid transport, is highly expressed in BAT, but not differentially.

Two phospholipases, *PLA2G4C* (phospholipase A2, group IVC) and *PLA2G16* (phospholipase A2, group XVI) are differentially expressed in BAT. *PLA2G4C* has a particularly dramatic rise in expression at the torpor timepoint (two-fold higher than IBA, three-fold higher than April). PLA2G4C is known to preferentially cleave arachidonic acid from the second phosphatidylcholine site [29]. It may be present in the mitochondria [30], where its long chain fatty acid products activate UCP1 [31]. It is thus interesting that SCGB1A1 (secretoglobin, family 1A, member 1), which is BAT-specific within our data, inhibits phospholipase A2 activity, suppresses inflammatory responses [32], and interacts with cubulin [33]. *SCGB1A1* mRNA levels are significantly higher in torpor and IBA compared to April and October.

**Figure 7:**
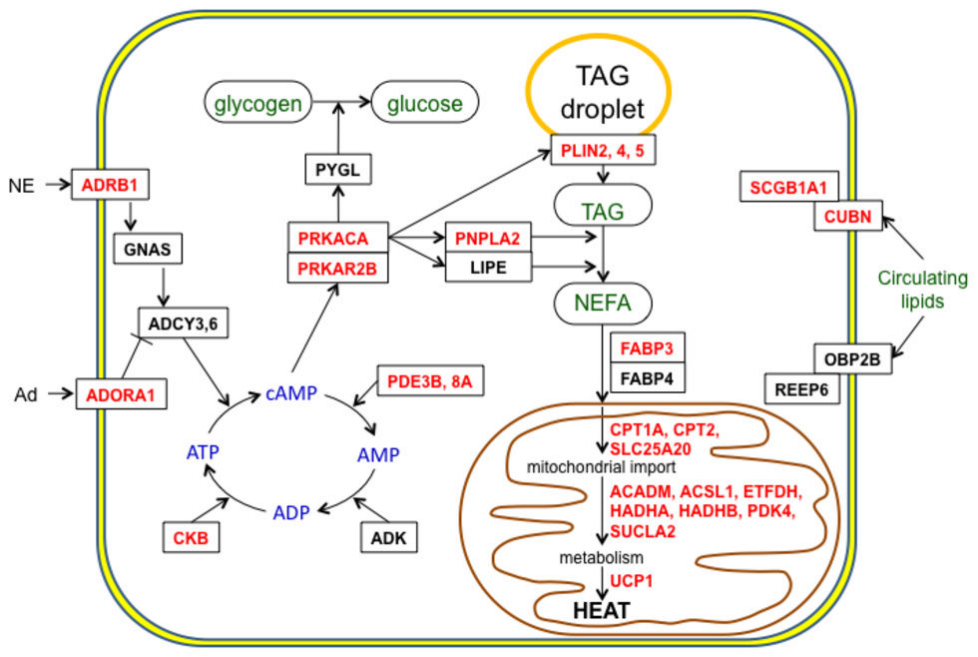
Model highlighting differentially expressed genes involved in fuel utilization and heat generation in BAT during hibernation. The role of the gene products in various metabolic processes in a brown adipocyte is shown. Genes with abbreviations in red meet the criteria for differential expression showing highest mRNA levels during the hibernation phases of torpor or interbout arousal. Abbreviations of genes that are not differentially expressed but are highly expressed, and/or tissue-specific genes in BAT, are shown in black. Molecules that serve as sources of fuel are labeled in green. Gene name abbreviations are defined in Table S1. Abbreviations: Ad, adenosine; NE, norepinepherine; NEFA, non-esterified fatty acids; TAG, triacylglycerol.

Figure 7 is a model showing the involvement of highly expressed and/or differentially expressed ground squirrel BAT genes involved in adrenergic signalling, lipolysis and heat generation during hibernation. 

### Transcription factors

One of the most highly expressed genes in BAT is *MEF2D*, a MADS-box transcription factor. *MEF2D* is not differentially expressed but is highly expressed in BAT versus other ground squirrel tissues that are available for comparison including WAT (Figure 3). MEF2D has recently been shown to positively regulate mitochondrial transcription [34], and is known to play a crucial role in regulating muscle-specific gene expression through histone modification [35].

**Figure 8:**
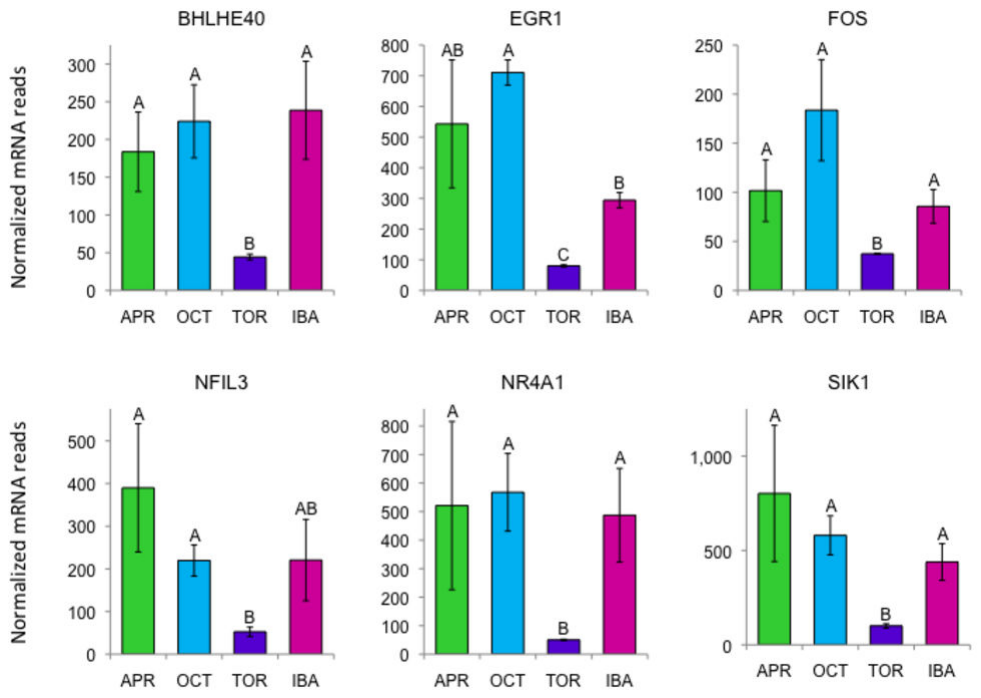
Differentially expressed genes encoding BAT transcription factor/regulator mRNAs that are low in torpor. Error bars represent standard error of the mean. Transcript levels for all genes shown have an FDR<0.05. The letters above each bar represent post hoc pair-wise comparisons to determine significance between collection points. Any collection point not connected by the same letter is significantly different (FDR<0.05). Abbreviations: *BHLHE40*, class E basic helix-loop-helix protein 40; EGR1, early growth response 1; *FOS*, FBJ murine osteosarcoma viral oncogene homolog; NFIL3, nuclear factor, interleukin 3 regulated; *NR4A1*, nuclear receptor subfamily 4, group A, member 1; SIK1, salt-inducible kinase 1.

 A number of differentially expressed transcription factors showed unusual patterns of expression in BAT. Many of them have very low expression at torpor only to rebound at IBA, suggesting they are involved in rapid changes in gene expression between these two phases of hibernation. Some of these genes are highlighted in Table 3 and Figure 8. One example is *NR4A1* (nuclear receptor subfamily 4, group A, member 1, also known as NUR77), which is known to be involved in the control of *UCP1* expression [36] and is transcriptionally activated by MEF2D [37]. The related *NR4A2* and *NR4A3* genes have similar expression patterns as *NR4A1* (very low in torpor) but do not meet our criteria for differential expression. Another differentially expressed transcription factor is BHLHE40 (class E basic helix-loop-helix protein 40), which plays a critical role in circadian rhythms [38]. 

**Table 3:** Transcription factors/regulators in BAT.

Gene	Differentially expressed in BAT	Torpor level significantly lower than IBA	IBA to Torpor fold change	Number of normalized reads			
				April	October	Torpor	IBA
RASD1	Yes	Yes	10.9	373	409	27	295
NR4A1	Yes	Yes	9.7	521	568	50	487
BHLHE40	Yes	Yes	5.4	184	224	44	239
JUN	Yes	Yes	4.8	343	392	89	425
SIK1	Yes	Yes	4.3	802	580	101	439
NFIL3	Yes	No	4.2	390	220	53	221
ATF3	Yes	Yes	4.0	47	67	45	180
EGR1	Yes	Yes	3.7	543	711	80	294
FOS	Yes	Yes	2.3	102	184	37	86
JUNB	Yes	Yes	2.0	147	184	41	82
TSC22D3	Yes	No	1.4	699	373	138	190
MEF2D	No	No	1.1	54501	72957	61852	67403

A constellation of other transcription factor genes, that show lower expression in torpor than IBA, include ATF3 (activating transcription factor 3), *NFIL3* (nuclear factor, interleukin 3 regulated), *FOS* (FBJ murine osteosarcoma viral oncogene homolog), *JUNB* (jun B proto-oncogene), and EGR1 (early growth response 1). Reduced expression of these genes has also been shown to be associated with reduction of inflammation in patients undergoing gastric bypass surgery and omentectomy [39]. Several of these same genes have also been associated with circadian rhythms and/or the CREB pathway. A transcriptional regulator showing reduced expression during torpor is salt-inducible kinase 1(SIK1; Figure 8). SIK1 has been shown to repress the CREB pathway, but when stimulated by cAMP it relocates to the cytoplasm [40], linking CREB to the adrenergic pathway.

### Genes with high April expression

**Figure 9:**
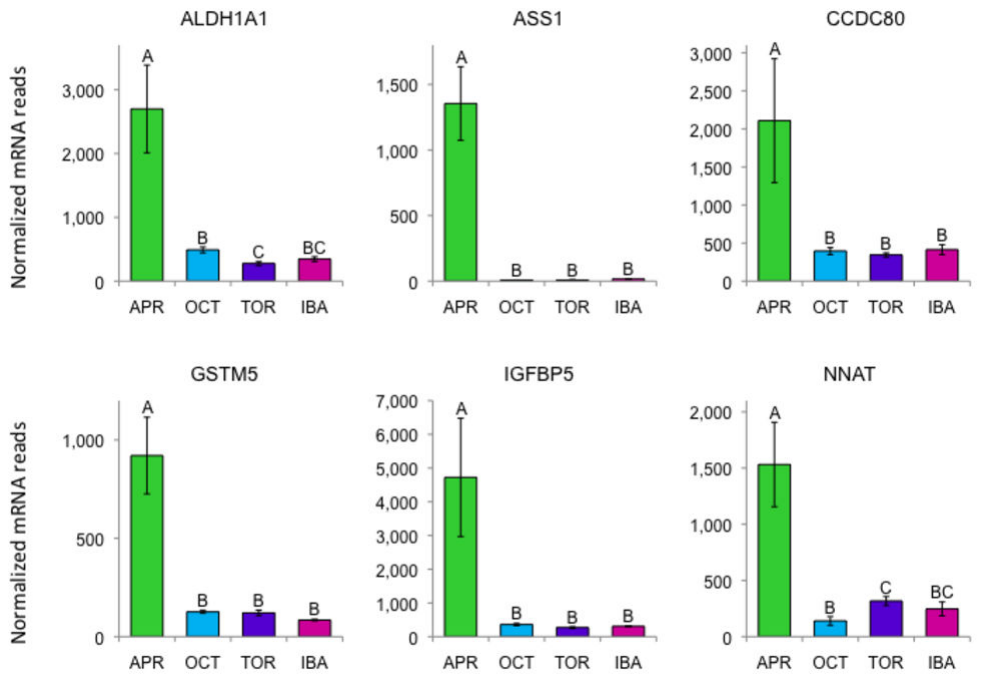
Differentially expressed genes with very high expression in April compared to all other collection points. Error bars represent standard error of the mean. Transcript levels for all genes shown have an FDR<0.05. The letters above each bar represent post hoc pair-wise comparisons to determine significance between collection points. Any collection point not connected by the same letter is significantly different (FDR<0.05). Abbreviations: ALDH1A1 aldehyde dehydrogenase 1 family member A1; ASS1, argininosuccinate synthase 1; *CCDC80*, coiled-coil domain-containing protein 80; *GSTM5* glutathione S-transferase mu 5; IGFBP5, insulin-like growth factor binding protein 5; NNAT, neuronatin.

Most of the data presented above focuses on genes that are more highly expressed at the torpor and IBA time points. The interpretation of expression patterns in BAT during hibernation is much easier because of the extreme demands of thermogenesis during arousals. However, many of the differentially expressed genes show their highest expression levels during April. It is more difficult to characterize the pathways and processes involving these gene products, but some of the more extreme cases are highlighted in Table 4 and Figure 9.

**Table 4:** Differentially expressed BAT genes showing highest expression in April.

Gene	Name/description	April to Torpor or IBA fold change	Number of normalized reads			
			April	October	Torpor	IBA
ASS1	argininosuccinate synthase 1	84.7	1355	8	9	16
FCN1	ficolin	64.5	5674	77	88	56
ACSF2	acyl-CoA synthetase family member 2	23.4	1711	93	68	73
BCAT2	branched chain amino-acid transaminase 2, mitochondrial	18.7	731	40	37	39
SLC1A5	solute carrier family 1	18.1	507	37	13	28
IGFBP5	insulin-like growth factor binding protein 5	15.3	4725	360	271	309
ALDH1A1	aldehyde dehydrogenase 1 family, member A1	7.8	2699	491	278	346
IGLL1	immunoglobulin lambda-like polypeptide 1	7.8	1452	61	16	187
GSTM5	glutathione S-transferase mu 5	7.6	921	126	121	85
IGLL5	immunoglobulin lambda-like polypeptide 5	6.9	1496	51	14	218
HP	haptoglobin	6.3	4732	1950	749	489

The most differentially expressed gene in our entire dataset is *ASS1* (argininosuccinate synthase 1) whose expression plummets 169-fold from the April to the October time point and stays low during hibernation. Less dramatically *ASL* (argininosuccinate lyase) is also significantly higher in April compared to the other time points. ASS1 is the first enzyme in the urea cycle that acts outside of the mitochondria, catalyzing the formation of arginino-succinate from citrulline and aspartate. ASL in turn converts the arginino-succinate to arginine. The low expression of these genes during the hibernation season may be important for repressing the urea cycle and perhaps for preserving metabolites in the citric acid cycle.

There are 63 ribosomal proteins which are more highly expressed in April compared to torpor and IBA time points. In striking contrast, the only ribosomal proteins with lower expression in April compared to any other time point were mitochondrial ribosomal proteins. Taken together these results form a consistent pattern of suppressed nuclear ribosome and enhanced mitochondrial ribosome production during the hibernation season. The function of other genes with higher expression in April is less clear. For example, *FCN1* (ficolin-1) is at least 64-fold higher in April compared to the other time points. The eleven genes with at least a 6-fold change from April to any other time point and at least 500 normalized read counts are shown in Table 4.

## Discussion

Understanding the optimization of fat catabolism and heat generation in BAT of the naturally hibernating thirteen-lined ground squirrel was the subject of this study. We performed deep sequencing of the BAT transcriptome at various times of the year to determine the identity and abundance of mRNAs that contribute to the hibernating phenotype.

### Regulation of BAT metabolism

Adrenergic signaling controls heat-generation in BAT. We found genes encoding the beta-adrenergic receptors ADRB1 and ADRB3 show opposite expression patterns during the year (Figure 5). The receptors are coupled to the G-protein stimulatory subunit (G_s_α) encoded by *GNAS*. Overall *GNAS* has the highest number of reads of any mRNA in BAT. Although our read counts are not corrected for the expected bias from the length of the transcript, the *GNAS* transcript is not unusually long (approximately 2000 bases). By examining the distribution of reads along the genomic region containing the *GNAS* locus we confirmed that virtually all of the reads come from the shorter, active form [41]. The massive disparity in transcript levels for *GNAS* (Figure 3) versus the beta-adrenergic receptors *ADRB1* and *ADRB3* (Figure 5) is consistent with stoichiometric results for the protein products [42]. *GNAS* is epigenetically imprinted in the mouse, and its expression is important in lipid clearance, energy metabolism, and thermogenesis [43-45].

Although several studies suggest that the alpha-2 (inhibitory) adrenergic receptors are present in BAT of some species, we found very few counts from *ADRA2A* in BAT (maximum of 6 counts in April). The absence of alpha-2 adrenergic receptors in other hibernating species (for example the golden hamster) provides some indirect evidence that the alpha-2 adrenergic receptors are not present in hibernator BAT in significant amounts [46]. The inhibitory pathway is transduced through the G-protein inhibitory proteins GNAI1, GNAI2, and GNAI3. *GNAI1* is differentially expressed in BAT with higher values in October, torpor, and IBA. There was some moderate expression of the inhibitory G-proteins *GNAI2* and *GNAI3*, although they were not differentially expressed. 

In order to quickly elevate internal body temperatures during an IBA, BAT must mobilize its TAG droplet lipid stores. Many genes involved in this process (perilipins, lipases, fatty acid binding proteins) are either differentially expressed or highly abundant (Figure 7). It is somewhat surprising that *PNLIP* (pancreatic triacylglycerol lipase) was expressed at low levels in BAT considering its lipolytic activity at cold temperatures and expression during hibernation in WAT and heart of this species [47-49]. In contrast, LPL (lipoprotein lipase) is higher in BAT during April, when the squirrels were fed *ad libitum*. 

After removal of two fatty acids from a triacylglcerol, the final fatty acid is cleaved from monoglycerides primarily by MGLL (monoglyceride lipase). *MGLL* and its coactivator *ABHD5* are strongly but not differentially expressed in BAT. After lipolysis some of the resulting glycerol probably leaves BAT through aquaporin 7 (*AQP7*), which is strongly expressed. AQP7 is known to translocate to the plasma membrane after adrenergic stimulation and to facilitate the flow of glycerol out of the cell [50]. However GK (glycerol kinase) is also strongly expressed in BAT, unlike WAT, indicating that some glycerol is further metabolized in BAT.

### Depolarization and ion flux

All cells maintain some concentration differences across their membranes which usually result in a membrane potential. For example, WAT has a resting potential of about -30 mV, while BAT is about -48 mV but can be -60 mV in the absence of bicarbonate [1]. Like neural and muscle cells, BAT can depolarize, although the purpose of this depolarization remains unknown. The depolarization of BAT occurs in at least three stages [1]: (A) a release of intracellular calcium, (B) a Cl^-^ current, and (C) Na^+^ and K^+^ currents.

We found a few differentially expressed genes in BAT that may relate to stage (*A*) above. *PLCD3*, phospholipase C, delta 3, is higher in torpor and IBA with moderately high expression. This enzyme hydrolyzes phosphatidylinositol 4,5-bisphosphate (PIP2) into diacylglycerol and inositol 1,4,5-trisphosphate (IP3). *PLCB4*, phospholipase C, beta 4, is moderately but not differentially expressed, as are *PLCL2*, phospholipase C-like 2, *PLCG1*, phospholipase C, gamma 1, and *PLCB3*, phospholipase C, beta 3. *CIB2*, calcium and integrin binding family member 2, which is very similar to calcineurin B and calmodulin, is differentially expressed with higher levels in October, torpor and IBA.

The chloride current, stage (*B*), may involve *CLIC5*, chloride intracellular channel 5, which is differentially expressed in BAT with highest expression in torpor and IBA. It may be notable that CLIC5 can also conduct Na^+^ and K^+^ currents [51]. These ion currents may facilitate the transport of carbon dioxide in the form of bicarbonate out of the cell therefore preventing acidosis. They could also explain the otherwise puzzling expression of the genes coding acid-sensing ion channels *ASIC1* and *ASIC4*, which are both more highly expressed during the hibernation season. In addition, the H^+^/K^+^ exchanging ATPase *ATP4A* shows an unusually extreme expression pattern, with very low values during the IBA time point compared to torpor. 

Depolarization could also be associated with endocytosis during arousal. *MYO5B* (myosin VB) is differentially expressed with low expression in April, and has a known role in membrane recycling [52]. In this context it is also worth noting the genes GJB1 (gap junction beta 1) and *CGNL1* (cingulin-like 1) are significantly lower in April. It has long been known that BAT adipocytes are linked by gap junctions [53], which presumably synchronize the depolarization throughout the tissue.

### Expression of neural and inner-ear related genes

*TECTB* (tectorin beta) was both differentially expressed and highly BAT-specific. This extracellular protein is known to be important in the tectorial membrane of the inner ear [54]. Other BAT-expressed genes associated with the inner ear include *OTOF* (otoferlin), *USH1C* (Usher syndrome 1 C), and *SLC12A7* (solute carrier family 12, member 7). *OTOF* is highly expressed in BAT during torpor and has been described to have a role in neurotransmitter release of cochlear inner hair cells [55]. It is possible that *OTOF* expression occurs in innervating axons of BAT rather than the brown adipocytes themselves (reviewed in [56]). *SLC12A7* is highly expressed in peripheral nerves and is important for the survival of cochlear outer and inner hair cells [57]. Its expression in BAT may also be from the innervating sympathetic nerves within the organ [1]. 

### Comparison with BAT in mice

With more than 75 million obese adults in the U.S., and a national obesity rate of 35% [58], the presence of BAT in humans has received considerable attention due to its unique ability to rapidly burn fat. Despite the excitement associated with BAT as a means to combat obesity, little is known about the molecular mechanisms for optimizing BAT function. Recently, an aspect of BAT research receiving considerable attention is the prospect of “browning” white fat to generate an efficient fat-burning “beige” or “brite” fat (reviewed in [59]). Studies in mice have emphasized the role of PPARGC1A (peroxisome proliferator-activated receptor gamma, coactivator-related 1) in this process [60,61]. We found the *PPARGC1A* gene to be differentially expressed in the BAT of ground squirrels, with the highest values during IBA (Table S2). 

The depth of transcriptomic detail provided by our study identified many of the genes involved in pathways for thermogenesis, lipolysis, beta-adrenergic activation, and also suggested novel patterns of gene activity. While some classic markers of BAT in mice such as *UCP1* and *CIDEA* are highly expressed in our ground squirrel samples, others such as *FGF21* [62], *PRDM16* [63], and *ZIC1* [64] are either absent at all collection points, or present only at low levels and not differentially expressed. It may be that *PRDM16* is most important in the initial development of BAT, and that its expression is not necessary in the adult ground squirrel. Several other highly BAT-specific genes in ground squirrels including *REEP6, OBP2B*, *TECTB*, *SCGB1A1*, do not show similar expression patterns in a comparison of BAT and WAT in mice [GEO dataset GDS2813]. 

## Materials and Methods

### Animals

The thirteen-lined ground squirrel, *Ictidomys tridecemlineatus*, is our model for mammalian hibernation. This species is abundant and unprotected in Minnesota where they are known to damage agricultural crops (http://www.dnr.state.mn.us/mammals/thirteenlinedgroundsquirrel.html). The squirrels were live-trapped on private property with permission near Paynesville, Minnesota by slowly pouring water into burrows and capturing the animal in a butterfly net when it emerges. Animals were housed in the designated animal facility at the University of Minnesota Medical School Duluth and all experimental procedures were approved by the University of Minnesota Institutional Animal Care and Use Committee (protocol #1103A97712).

Following capture squirrels are kept individually in plastic top-load rat cages filled with aspen shavings. Diet consists of standard rodent chow (Purina, #5001) and water *ad libitum*. A total of 12 male and 12 female thirteen-lined ground squirrels were used in these experiments. The squirrels were kept at room temperature in a 12:12 light/dark cycle at 23 °C and fed standard rodent chow and water *ad libitum* from April through October. Cardboard tubes from paper towel rolls were provided as enrichment from April through October. During the hibernation season (November-March), the squirrels were moved into an artificial hibernation chamber and kept in constant darkness at 5-7°C with no food provided. All animals were deeply anesthetized with 5% isoflurane and killed by decapitation to minimize pain and discomfort at the time of sacrifice.

### Collection points

The collection points for these experiments were chosen to uncover the most meaningful comparisons in ground squirrel BAT across the hibernation season. Four collection points were used: pre-hibernation (October active), torpor, IBA, and post-hibernation (April active) (Figure 1). Three males and three females were sacrificed at each collection point. Animal state at each point was verified by rectal body temperature and animal behavior (Torpor: 6-8ºC/inactive; Active and IBA: 35-37ºC/active). All animals were sacrificed between 10 am and 3 pm. 

Visceral white adipose tissue (WAT) was dissected to collect only the retroperitoneal fat pad [65]. BAT samples were taken from the axillary pad. The axillary BAT pad is located in the left and right axilla along the lateral aspect of the rib cage between the musculature of the anterior and posterior axillary folds.  The BAT pads are dissected from the surrounding tissues, cleaned of any contaminating tissue, and flash frozen in liquid nitrogen. Three samples were sequenced for each time point. Each sequenced sample used RNA pooled from two individuals, one male and one female.

### Illumina sequencing of phase-specific BAT RNA

Total BAT RNA was isolated from ground squirrels at the following four times/activity states: April active; October active; January torpor; January IBA. RNA was purified by standard procedures used in our lab [10]. Generation of the ground squirrel BAT transcriptome was performed at the Biomedical Genomics Center (BMGC) at the University of Minnesota Twin Cities. Total RNA integrity was assessed using capillary electrophoresis (e.g., Agilent BioAnalyzer 2100), generating an RNA Integrity Number (RIN). To pass the initial Quality Control (QC) step, at least 1 microgram of BAT RNA required a RIN of 8 or greater before it is converted to Illumina sequencing libraries.

### Library creation

From each BAT replicate 1 microgram of total RNA (equal RNA from 1 female and 1 male) was converted to poly(A)+ RNA using oligo-dT coated magnetic beads, fragmented and then reverse transcribed into cDNA. The cDNA is fragmented, blunt-ended, and ligated to indexed (barcoded) adaptors and amplified using 15 cycles of PCR. Final library size distribution was validated using capillary electrophoresis and quantified using fluorimetry (PicoGreen) and by Q-PCR.

### Cluster generation and sequencing

Truseq libraries were hybridized to a paired-end flow cell and individual fragments were clonally amplified by bridge amplification on the Illumina cBot. Libraries were clustered at a concentration of 12pM. Once clustering was complete, the flow cell was loaded on the HiSeq 2000 and sequenced using Illumina’s SBS chemistry. Upon completion of a read, a 7 base pair index read is performed. Samples were run for 100 cycles with a minimum of 10 million single reads per sample.

Base call (.bcl) files for each cycle of sequencing were generated by Illumina Real Time Analysis (RTA) software. The base call files and run folders are then exported to servers maintained at the Minnesota Supercomputing Institute. Primary analysis and de-multiplexing are performed using Illumina’s CASAVA software 1.8.2, resulting in de-multiplexed FASTQ files.

### Bioinformatic analysis

Each Illumina HiSeq read was 100 bases. BAT sequences were mapped to contigs constructed from other Illumina reads from this project and from brain cortex and hypothalamus [10], and skeletal muscle, heart, and white adipose tissue RNA sequence data recently determined by the Andrews Lab. A preliminary set of contigs was assembled by the Trinity suite of programs [11].  Trinity was also used to predict coding domain subsequences from these contigs.  Any contig containing a predicted coding domain was then trimmed to this domain plus up to 100 bases on both ends of the domain.  These sequences were then compared to the NCBI RefSeq human mRNA sequences using NCBI Blastn [12]. Before matching reads to the contigs, mitochondrially-encoded reads were screened using the thirteen-lined ground squirrel mitochondrial genome sequence [13] and NCBI's megablast program [12] due to the high density of mitochondrial genomes in BAT. Genes encoding 835 nuclear-encoded mitochondrial proteins were identified using annotations by NCBI for RefSeq entries and using Gene Ontology gene associations [66]. Original sequence data is available from the NCBI Sequence Read Archive under accession SRS396817. 

### Differential gene expression

The data used to determine if a gene was differentially expressed across the hibernation season consisted of the counted number of RNA sequences that were positively identified with each identified gene in each sample.  Samples consisted of three replicates of each of the following four phases: April active; October active; January Torpor; January IBA.  Each replicate consists of RNA extracted from one male and one female ground squirrel.  Replicates are true replicates and not repeated measures because different individuals were included in each group.  Data was normalized to the upper-quartile across all BAT samples [67].

### Analysis by DESeq

Differential gene expression was estimated using DESeq v1.6.1, an R program language package available through the Bioconductor platform [68]. DESeq estimates the variance-mean dependence in count data from high-throughput sequencing assays and tests for differential gene expression [69]. Upper-quartile normalized sequence counts for each gene in each sample were compiled in a tab-delimited text document that was read into R v2.14.1 (copyright 2004-2011, The R Foundation for Statistical Computing). Other than using upper-quartile normalization, analysis proceeded as described by Anders and Huber [69]. 

## Supporting Information

Table S1 **Distinct transcripts in ground squirrel BAT that mapped to human mRNAs.** Table provides the mean (n=3) and standard error for each of the four collection points of all 14,573 genes that are expressed in brown adipose tissue. Gene names listed are the official HGNC and human UNIPROT designations. APR: April collection point; IBA: Interbout arousal collection point; OCT: October collection point; TOR: Torpor collection point; std. error: standard error.
(XLS)

Table S2 **Differentially expressed BAT genes.** Table provides the mean (n=3) and standard error for each of the four collection points of all 2,083 genes that are differentially expressed in brown adipose tissue. The p-value generated from the DESeq analysis of deviance is also provided for each gene. A gene was considered differentially expressed and included in this Table if at least one collection point had at least 100 total counts, there was at least a 50% change between the collection points with the highest and lowest counts, and the FDR was less than 0.05. The p-value cutoffs for significance (FDR<0.05) was obtained from the Benjamini-Hochberg method. Gene names listed are the official HGNC and human UNIPROT designations. APR: April collection point; IBA: Interbout arousal collection point; OCT: October collection point; TOR: Torpor collection point; std. error: standard error.
(XLS)
